# Children's psychological traits and educational performance: How schools and residential areas moderate how individual traits translate into academic outcomes

**DOI:** 10.1002/jcv2.70100

**Published:** 2026-02-04

**Authors:** Qi Qin, Rosa Cheesman, Espen Moen Eilertsen, Eivind Ystrom

**Affiliations:** ^1^ Department of Psychology PROMENTA Research Center University of Oslo Oslo Norway; ^2^ PsychGen Centre for Genetic Epidemiology and Mental Health Mental Health and Quality of Life Norwegian Institute of Public Health Oslo Norway; ^3^ Centre for Research on Equality in Education University of Oslo Oslo Norway

**Keywords:** educational performance, environmental moderation, multilevel modeling, psychological traits

## Abstract

**Background:**

The extent to which children's psychological traits influence their educational performance is thought to depend on the fit between the individual and their developmental context. However, this assumption has yet to be empirically tested on a population scale. This study examines how neurodevelopmental, mental health, and personality traits interact with latent environmental contexts to enhance, maintain, or mitigate their educational performance.

**Methods:**

Using the Norwegian Mother, Father and Child Cohort Study (MoBa), we estimated the association between 16 developmental traits and grade point average (GPA) in 26,875 children across environmental contexts in 2131 primary schools, 1075 middle schools, ~14,000 neighborhoods, 1471 districts, and 347 municipalities.

**Results:**

Across the developmental traits, the effect on GPA ranged from −0.286 to 0.220. However, these effects varied substantially across environmental contexts (SD 0.022 to 0.090). Trait‐by‐context interactions were detected in primary schools (2 traits), middle schools (6 traits), neigbourhoods (0 traits), districts (3 traits), and municipalities (1 trait), respectively. Moderation magnitudes were largest for neurodevelopmental traits in primary schools. On average, the effects of ADHD and communication difficulties were −0.252 and −0.193. However, these effects differed substantially across primary schools with SD 0.090 and 0.070, implying *b* < −0.370 and *b* < −0.282 in 10% of schools. For average children, primary schools explained 0.504% of middle school GPA. Similarly, the effect of depression and extraversion was dependent on middle‐school environments (*b* = −0.128; SD = 0.058 and *b* = 0.024; SD = 0.022). Moreover, schools and districts with high GPAs tended to express lower detrimental effects on children's developmental traits.

**Conclusion:**

Schools and residential areas function as educational catalysts, enhancing or mitigating the influence of developmental traits. High‐performing environments compensate for psychological challenges, whereas lower‐performing contexts exacerbate difficulties. Because the study integrated multiple traits and contextual levels, the analyses were exploratory and data‐driven, and the findings should be interpreted as hypothesis‐generating. These results suggest a need for both context‐sensitive and individually tailored educational interventions.

## Key Points

**What's known?**

- Schools are known to moderate the relationship between ADHD and academic achievement, but it remains unclear whether residential areas also play a similar role or whether this applies to other psychological traits.

**What's new?**

- This study examines how children's psychological traits influence academic performance across primary and middle schools, districts, and municipalities, revealing that schools have the strongest moderating effect on grade point average (GPA).
- The findings highlight compensatory effects in high‐performing environments, where children with psychological challenges achieve at levels comparable to their peers with fewer challenges.

**What's relevant?**

- These results emphasize the need for context‐specific educational interventions tailored to children's psychological traits.

## INTRODUCTION

Educational performance is a key indicator in children's developmental trajectories, influencing future life outcomes such as access to higher education, financial wellbeing, and social mobility (Barnett & Belfield, 2006; Furnham & Cheng, 2017; Kern & Friedman, 2008). Understanding what factors have shaped the variation in children's educational performance at childhood remains essential for developmental and policy implications. While individual cognitive skills are vital to educational success, a variety of non‐cognitive psychological traits such as neurodevelopmental disorders (e.g., attention deficit hyperactivity disorder, autism spectrum disorder), mental health issues (e.g., anxiety, depression, conduct disorders), and personality traits also significantly impact children's educational performance (Agnafors et al., 2021; Brännlund et al., 2017; Dias et al., 2022; Hinshaw, 1992; Nadeau et al., 2020; Nordmo et al., 2022; Shiner & Caspi, 2003). These traits affect children's motivation, concentration, learning and coping capacity, with early internalizing and externalizing problems that predict later educational and social outcomes (Bolger, 1990; Daley & Birchwood, 2010; Ende et al., 2016; Kroneman et al., 2004; Loe & Feldman, 2007; Rapport et al., 2001).

Psychological traits, however, do not shape children's educational performance in isolation. Their influences on educational performance are context‐dependent, shaped by the individual's surroundings over time, encompassing their interrelated families, schools, neighborhoods, and broader society (Braveman & Gottlieb, 2014; Bronfenbrenner & Morris, 2007; Craik, 1973; Leventhal & Brooks‐Gunn, 2000; Ogden & Hagen, 2018). Schools and residential areas where children study and live daily, in particular, amplify or mitigate the effect of psychological traits on educational performance (Reiss et al., 2013; Shanahan & Hofer, 2005). For example, a school environment with special needs resources and teacher sensitivity might buffer the adverse effects of ADHD on educational performance, whereas a poor school environment may exacerbate these difficulties. Research indicates that in better‐performing schools, the impact of psychological disorders such as ADHD on educational attainment is often weaker due to school compensatory support (Cheesman, Borgen, et al., 2022; Cheesman, Eilertsen, et al., 2022). Children in disadvantaged neighborhoods also experience more behavioral and mental issues that have negative effects on educational performance (Borgen & Zachrisson, 2024; Flouri & Sarmadi, 2016; Nieuwenhuis & Hooimeijer, 2016; Ruiz et al., 2018).

Despite these insights, prior research has often been limited in scope. Many studies examine isolated environmental factors (e.g., school or family structure) without fully accounting for the interconnected systems in which children develop (Nieuwenhuis & Hooimeijer, 2016; O’Malley et al., 2015). Moreover, the extent to which these environments moderate the effects of various psychological traits remains insufficiently explored. While some evidence suggests that disadvantaged schools or neighborhoods exacerbate the impact of mental health problems, the specific mechanisms and the differential effects on diverse psychological traits are not well understood (Flouri & Sarmadi, 2016; Kroneman et al., 2004). To better support children with specific psychological barriers, it is paramount to map out how traits are differentially transacted across environments to identify which school or residential contexts most effectively support psychologically diverse children and clarify how different contexts moderate these associations.

This study examines how key environmental contexts, such as schools and residential areas, interact with the variations in children's psychological traits to influence educational performance. Focusing on a Norwegian context, we investigate whether the associations between psychological traits and educational performance differ across schools and neighborhoods. By identifying the environments that may buffer or amplify the effects of psychological difficulties, the current study provides insights into how contextual differences contribute to educational disparities and how schools and communities can better support psychologically diverse children. Given the integration of multiple traits and contextual levels, the present study was designed as an exploratory, data‐driven investigation rather than a preregistered hypothesis test, and the findings should therefore be interpreted as hypothesis‐generating.

By examining how individual psychological traits interact with environmental factors, this study contributes to understanding how school and residential contexts relate to children's educational performance. Identifying environments that may buffer or amplify the effects of psychological difficulties offers insight into how contextual differences contribute to educational disparities. The findings may also inform efforts in educational and clinical settings to support children with diverse psychological profiles.

## METHODS

### The Norwegian context

Norway is characterized by an egalitarian social democratic welfare system with low wealth inequality and great public support in health, education, and social protection (Barth et al., 2021). Universal healthcare and free compulsory education in primary and middle schools are provided to nearly all children regardless of their residency across the country. Children with mental health concerns access assistance through school health services, local general practitioners, mental health services in the local municipalities. Adapted special needs education in addition to psychopharmacological treatments defined by the national guidelines are considered the pivotal interventions.

The municipalities take the responsibility for running the primary and middle schools, as well as providing before and after school care for all students between grades 1–4 and for students with special needs from grades 1–7. Students are not graded with marks in primary school (grade 1–7, age 6–12), and are only graded on mandatory courses each school year in the middle school (grade 8–10, age 13–15). These marks are not used for structural streaming in compulsory education, but as a measure for the evaluation entitling students to non‐compulsory upper secondary education. Despite the sound Nordic welfare and education system, a trend of increasing inequality is inevitably observed in economic, health, and education challenged by global competition and domestic discourses (Barth et al., 2021; Braathe & Otterstad, 2014; Dahl & van der Wel, 2015).

### Sample

This study uses data from the Norwegian Mother, Father, and Child Cohort Study (MoBa) linked to the Norwegian National Educational Database, and Statistics Norway (SSB). MoBa is a prospective population‐based pregnancy cohort study with a family design conducted by the Norwegian Institute of Public Health (Magnus et al., 2016). Pregnant women who routinely attended the ultrasound examination were recruited from across Norway from 1999 to 2009, with a participation rate of 41%. The cohort includes 114,500 children, 95,200 mothers, and 75,200 fathers. Blood samples from both parents and children were taken, and questionnaires on various questions regarding health and lifestyle exposures were completed by these participants both during and after the pregnancy.

Our study focuses on a subsample of 26,875 children whose mothers completed psychological trait questionnaires when the children were 8 years old (Supporting Information S1: Figure S1). The records were then further linked with the administrative data on children's educational performance obtained at the end of the middle school, as well as the administrative identifiers for school memberships and geographical identifications such as municipalities, districts, and neighborhoods. We kept a unique identifier for each child in the datasets. Because no validated methods exist for imputing cluster membership variables in multilevel models, children lacking valid school or geographical codes could not be reliably assigned to contextual units and were therefore excluded. The final analytic sample thus consists of participants with complete linkage across psychological measures, educational records, and contextual identifiers.

### Measures

#### Psychological traits

As shown in Table 1, a total of 16 traits related to children's psychological development from 8 scales of the MoBa 8‐year‐old questionnaire were included in the current study (Supporting Information S1: Figure S2). These traits were chosen to represent the main domains of child psychological functioning, including externalizing behavior, internalizing issues, autism‐spectrum disorders, and personality traits that are known to influence learning and educational performance. Responded by mothers, this questionnaire focuses on children's social and mental health development among all the MoBa questionnaires and measures mental health development during children's compulsory education using psychometrically established instruments. Reversed responses were recoded, and a sum score for each child was created by summing items across each scale. The observations were excluded if there was any missing data in the item response. The sum scores are group mean centered with a mean of 0 and a standard deviation of 1 (Supporting Information S1: Figure S3). The 5‐point scale Norwegian Hierarchical Personality Inventory for Children was answered by mothers to measure children's personality traits (NHiPIC‐30; Vollrath et al., 2016). Notably, versions A and B&C were implemented in the data collection process with changes in the items. In this study, we applied item response theory (IRT; Baker, 2001) models to harmonize the three versions by calculating the latent IRT scores for each personality trait in each child.

**TABLE 1:** Psychological traits and scales included in the study.

Domain	Trait	Scale	Reference
Externalizing/disruptive behavior disorders	Attention‐deficit/hyperactivity disorder (ADHD)	Parent/Teacher Rating Scale for Disruptive Behavior Disorders (RS‐DBD)	Silva et al. (2005)
	Attention‐deficit (AD)		
	Hyperactivity disorder (HD)		
	Conduct disorder (CD)		
	Oppositional defiant disorder (ODD)		
Internalizing disorders	Depression	Short Mood and Feelings Questionnaire (SMFQ)	Sharp et al. (2006)
	Anxiety	Screen for Child Anxiety‐Related Disorders (SCARED)	Birmaher et al. (1999)
	Eating disorder	Children's Eating Behavior Questionnaire (CEBQ)	Wardle et al. (2001)
Autism spectrum disorder	Autism	Social Communication Questionnaire (SCQ)	Rutter, et al. (2003)
	Communication skills	Children's Communication Checklist‐2 (CCC‐2)	Norbury et al. (2004)
	Language difficulties	Checklist of 20 Statements about Language‐Related Difficulties (Språk20)	Ottem (2009)
Personality traits	Extraversion	Short Norwegian Hierarchical Personality Inventory for Children (NHiPIC‐30)	Vollrath et al. (2016)
	Agreeableness		
	Conscientiousness		
	Neuroticism		
	Openness		

#### School performance

The outcome variable school performance was measured by the student's grade point average (GPA/Grunnskolepoeng) downloaded from the Norwegian National Education Database (2001–2022). The students' GPAs are obtained at the end of the compulsory education grade 10 age 15–16, and form the basis for admission to upper secondary school. Scores from each subject were rated by teachers at school, thus it reflects more than academic knowledge and exam skills but also the comprehensive abilities of students, potential teacher‐student relationships, and teacher biases. The GPA is an average score calculated from all the subject scores listed on the diploma times 10, ranging from 10 to 60 (Supporting Information S1: Figure S4). Higher scores represent greater competence. In the current study, we excluded the cases with a GPA score of 0 or higher than 60 and the cases where the GPA is missing. If duplicated GPA records for the same student exists, the latest score was retained.

#### Family, school, and geographical identifiers

Family identifiers were extracted from the Norwegian national registry. School identifiers for both primary and middle schools were extracted from the National Education database.

Geographical identifiers from Statistics Norway include municipality (kommune; *n* = 357), district (delområde; *n* = 1550), and neighborhood (grunnkretser; *n* ˜ 14,000) are linked to students' records. The municipalities are divided into stable basic adjacent geographical units neighborhood, covering consistent numbers of inhabitants (approximately 350) living in homogeneous conditions. The neighborhoods are further aggregated into sub‐areas called districts in terms of natural geography and communication. The district is an intermediate level between municipality and neighborhood, which avoids over‐detailed information while providing an overview of certain areas. Children usually attend schools near home, however, occasionally children from one neighborhood may attend different schools, and children in the same school might live in different neighborhoods.

Administrative divisions change over time due to policy adjustments. Using the norgeo R package, we harmonized geographical identifiers from 1990 to 2022, resolving changes in municipal and district codes. However, it is difficult to track the splits of administrative areas. For example, in 2018, the neighborhood Nydalen (code 03014404) has been split into neighborhood Nydalen vest (code 03014419) and Nydalen øst (code 03014420). It is unknown whether and which children in Nydalen in 2017 were divided into Nydalen vest or Nydalen øst in 2018. Also, it is challenging to harmonize the identifiers at the neighborhood level due to anonymity, but stable neighborhood identifiers ensure consistency.

### Statistical analyses

To disentangle how children's educational performance predicted by their psychological traits interacts with the surrounding environments, we applied a total of 112 linear mixed models (Demidenko, 2013; Rabe‐Hesketh & Skrondal, 2008) in the current study. Among them, a series of 7 models were specified for each of the 16 psychological traits separately, incorporating 6 levels: family, primary school, middle school, neighborhood, district, and municipality. The models can be summarized in the equation below:

$${\text{GPA}}_{\text{ifpmndk}}=\beta_{1}+\beta_{2}X_{\text{ifpmndk}}+\eta_{0F}+\eta_{0p}+\eta_{1p}X_{\text{ifpmndk}}+\eta_{0m}+\eta_{1m}X_{\text{ifpmndk}}+\eta_{0n}+\eta_{1n}X_{\text{ifpmndk}}+\eta_{0d}+\eta_{1d}X_{\text{ifpmndk}}+\eta_{0k}+\eta_{1k}X_{\text{ifpmndk}}+\varepsilon_{\text{ifpmndk}},$$

where educational performance was explained by each psychological trait in clusters at individual level *i*, family level *f*, primary school level *p*, middle school level *m*, neighborhood level *n*, district level *d*, and municipality level *k* respectively. Fixed intercept is specified as *β*_0_, fixed slope *β*_1_, random intercept *η*_0_, random slopes *η*_1_, and the residual *ϵ*.

For each of the psychological traits, we first specified a baseline model where the psychological trait explains variations in educational performance. We allowed for the fixed effect for the psychological trait and random intercepts at the individual children's level.

Second, building upon the baseline model, we allowed for the random intercepts at family, primary school, middle school, neighborhood, district, and municipality levels for each psychological trait. Children are cross‐classified to the families, primary schools, middle schools, and residential areas. Neighborhoods are nested within districts, and districts are nested within municipalities.

Third, we tested for person‐environment interactions by allowing the slopes between children's psychological traits and their educational performance to vary across groups in different environmental contexts. We added the random slopes at each level increasingly as shown in Table 2. Based on the best‐fitted random intercept models, random slopes at primary school, middle school, district, and municipality levels were added to the model for each trait with all possible combinations of environmental contexts. Family level random slopes were excluded to focus on broader environmental influences and ensure model identification.

**TABLE 2:** Model specifications.

Model	Fixed effect	Random intercepts	Random slopes
1. Baseline	GPA (Intercept)	Family Primary school Middle school Neighborhood District Municipality	None
2–6. Between‐context educational performance differences	GPA (Intercept)	Permutation of any 5 of the random intercepts in the baseline model above	None
7–21. Between‐context educational performance differences	GPA (Intercept)	Permutation of any 4 of the random intercepts in the baseline model above	None
22–41. Between‐context educational performance differences	GPA (Intercept)	Permutation of any 3 of the random intercepts in the baseline model above	None
42–56. Between‐context educational performance differences	GPA (Intercept)	Permutation of any 2 of the random intercepts in the baseline model above	None
57–62. Between‐context educational performance differences	GPA (Intercept)	Each contains 1 random intercept from the baseline model	None
63. Trait‐by‐context interaction	Psychological traits (intercept &slope)	Family Primary school Middle school District Municipality	Primary school Middle school District Municipality
64–67. Trait‐by‐context interaction	Psychological traits (intercept &slope)	Family Primary school Middle school District Municipality	Permutation of any 3 of the random slopes in model 63
68–73. Trait‐by‐context interaction		Family Primary school Middle school District Municipality	Permutation of any 2 of the random slopes in model 63
74–77. Trait‐by‐context interaction	Psychological traits (intercept &slope)	Family Primary school Middle school District Municipality	Each contains 1 random slope from model 63
78. Trait‐by‐context interaction	Psychological traits (intercept &slope)	Family Primary school Middle school District Municipality	None

*Note*: The psychological traits refer to ADHD, AD, HD, CD, ODD, Depression, Anxiety, Eating disorder, Autism, Communication skills, Language impairment, Extraversion, Agreeableness, Conscientiousness, Neuroticism, and Openness. We ran the model series separately for each of these traits to avoid spurious correlations.

### Model fitting and comparisons

Maximum likelihood estimation (Haynes, 2013) was used to estimate the parameters in the model fitting procedure. We compare the fitted models gradually using the Akaike Information Criteria (AIC; Bozdogan, 1987)through the *r* function anova (). As a goodness of fit index, Akaike Information Criterion (AIC) estimates the model quality and handles the tradeoff between model fit and model complexity without overfitting. The lower AIC values indicate a better quality of the model fit. Because AIC already penalizes the inclusion of additional parameters and the models are nested and non‐independent, we did not apply a separate multiple‐testing correction across the fitted models. Analyses in the present study were all conducted in R software. Mixed models were fitted using the lme4 package (Bates et al., 2015).

## RESULTS

### Descriptive statistics

The sample consists of 26,875 students, from 24,887 families living in 8047 neighborhoods. Children were cross‐classified into 2131 primary schools, 1075 middle schools, 1471 districts, and 347 municipalities in Norway. On average, students per unit were: 77 per municipality (SD = 172.16), 18 per district (SD = 21.33), and 3 per neighborhood (SD = 3.59). Each family had approximately 1.08 children (SD = 0.28). Children who reside in the same district attend up to 16 different middle schools and 20 different primary schools. The number of children per primary school on average is 13 (range:1–70), and 25 for middle school (range:1–133).

Students' educational performance at the age of 16 (GPA) shows a slightly left‐skewed distribution with a skewness of −0.51 and a kurtosis of 3.1. The mean GPA in the sample is 45.6, and the standard deviation is 7.4. Full numbers of children in each grouping unit are provided in the supporting information (Supporting Information S1: Figure S5).

### Statistical analyses

We used a two‐step multilevel modeling approach to understand how school and residential environments moderate the relationship between psychological traits and GPA.

#### School and geographical variability in GPA

First, we established the environmental structure to quantify the school and geographical variability in children's GPA. The first set of model selection therefore determines whether and which level of environmental contexts should be kept in the model. Results showed that model five outperformed the rest of the models in model selection with lower goodness of fit indices (AIC; Table 3). In this model, random intercepts were specified at Family, Primary school, Middle school, District, and Municipality levels, however, the neighborhood level did not explain a significant variance in GPA.

**TABLE 3:** Model comparisons for identifying the environmental levels contributing to GPA variation.

Model	Constructed environmental contexts (random intercepts)	AIC
**Baseline model**	**Primary school, middle school, neighborhood, district, municipality, family**	**65750.70**
Model 1	Primary school, neighborhood, district, municipality, family	65791.82
Model 2	Middle school, neighborhood, district, municipality, family	65751.33
Model 3	Primary school, middle school, neighborhood, district, family	65867.42
Model 4	Primary school, middle school, neighborhood, municipality, family	65752.96
**Model 5**	**Primary school, middle school, district, municipality, family**	**65750.08**
Model 6	Primary school, middle school, neighborhood, district, municipality	66029.84
Model 7	Primary school, middle school, district, municipality	66039.21
Model 8	Primary school, middle school, neighborhood, municipality	66031.29
Model 9	Primary school, middle school, municipality, family	65753.49
Model 10	Primary school, middle school, neighborhood, district	66140.01
Model 11	Primary school, middle school, district, family	65866.67
Model 12	Primary school, middle school, neighborhood, family	65882.56
Model 13	Middle school, neighborhood, district, municipality	66031.11
Model 14	Middle school, district, municipality, family	65751.93
Model 15	Middle school, neighborhood, municipality, family	65757.70
Model 16	Middle school, neighborhood, district, family	65868.12
Model 17	Primary school, neighborhood, district, municipality	66073.16
Model 18	Primary school, district, municipality, family	65791.49
Model 19	Primary school, neighborhood, municipality, family	65805.63
Model 20	Primary school, neighborhood, district, family	65954.11
Model 21	Neighborhood, district, municipality, family	65801.44
Model 22	Middle school, primary school, municipality	66043.69
Model 23	Middle school, primary school, district	66148.75
Model 24	Middle school, primary school, neighborhood	66153.14
Model 25	Middle school, primary school, family	65884.21
Model 26	Middle school, municipality, district	66044.46
Model 27	Middle school, municipality, neighborhood	66036.72
Model 28	Middle school, municipality, family	65762.95
Model 29	Middle school, district, neighborhood	66141.36
Model 30	Middle school, district, family	65868.52
Model 31	Middle school, neighborhood, family	65892.04
Model 32	Primary school, municipality, district	66083.20
Model 33	Primary school, municipality, neighborhood	66085.37
Model 34	Primary school, municipality, family	65807.51
Model 35	Primary school, district, neighborhood	66227.80
Model 36	Primary school, district, family	65953.69
Model 37	Primary school, neighborhood, family	66034.67
Model 38	Municipality, district, neighborhood	66084.57
Model 39	Municipality, district, family	65805.31
Model 40	Municipality, neighborhood, family	65846.71
Model 41	District, neighborhood, family	65969.17
Model 42	Middle school, primary school	66167.69
Model 43	Middle school, municipality	66060.69
Model 44	Middle school, district	66153.84
Model 45	Middle school, neighborhood	66163.49
Model 46	Middle school, family	65900.92
Model 47	Primary school, municipality	66100.63
Model 48	Primary school, district	66237.15
Model 49	Primary school, neighborhood	66034.67
Model 50	Primary school, family	66040.08
Model 51	Municipality, district	66105.07
Model 52	Municipality, neighborhood	66129.01
Model 53	Municipality, family	65875.71
Model 54	District, neighborhood	66245.23
Model 55	District, family	65973.88
Model 56	Neighborhood, family	66223.44
Model 57	Middle school	66193.34
Model 58	Primary school	66322.18
Model 59	Municipality	66192.48
Model 60	District	66266.72
Model 61	Neighborhood	66488.69
Model 62	Family	66378.94

*Note*: This table presents the Akaike Information Criterion (AIC) values for 63 multilevel models, which differ in their random‐intercept structures. Each model includes a psychological trait as a fixed effect but varies in the combination of environmental contexts (family, primary school, middle school, neighborhood, district, municipality) specified as random intercepts. Lower AIC values indicate better model fit while penalizing model complexity. Model 5, which includes random intercepts for family, primary school, middle school, district, and municipality—but not neighborhood—shows the lowest AIC and therefore provides the best‐fitting representation of the environmental structure underlying variation in GPA. Corresponding BIC values and log‐likelihood statistics for all models are reported in Supporting Information S1: Table S1.

#### Trait effect on GPA by schools and residential areas

Second, we explored the extent to which the effects of children's traits make a difference in their educational performance between these environmental contexts. By adding the random slopes to model 5, the second set of models examined the heterogeneity in the effect of children's psychological traits on their GPA bwtween environments.

##### Main finding 1: Trait effects are moderated more by school than residential environments

**FIGURE 1:**
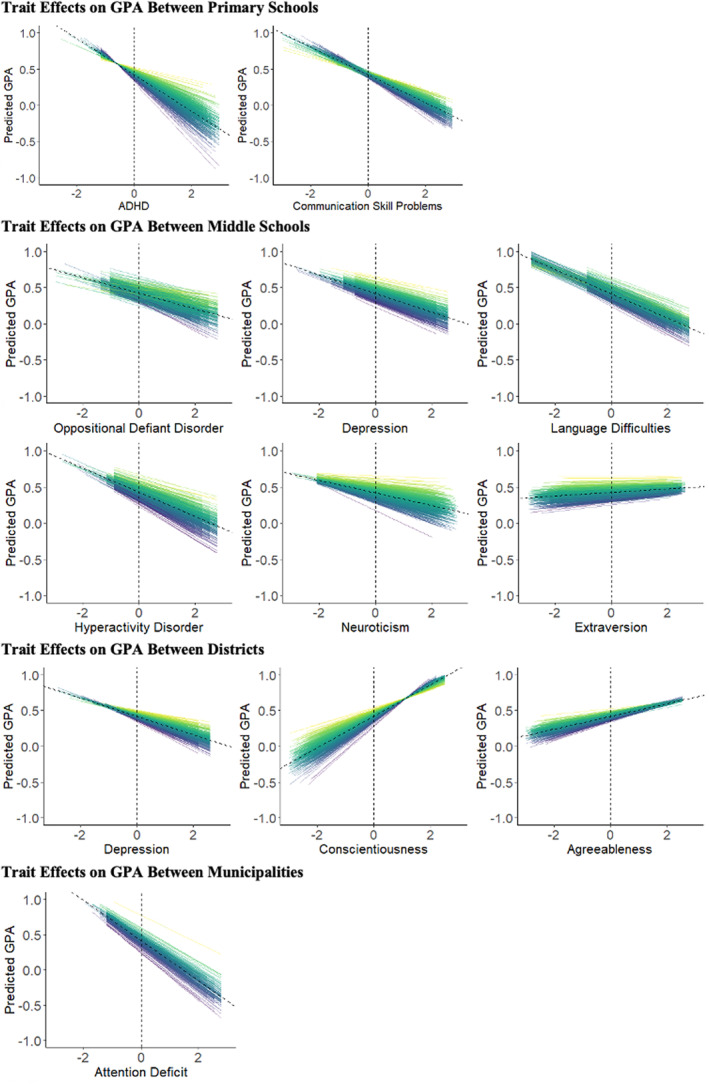
Trait Effects on GPA between Schools and Residential Areas. Each panel shows the predicted association between a psychological trait (*x*‐axis; standardized mean = 0, SD = 1) and students' Grade Point Average (GPA; *y*‐axis; standardized from the original 10–60 GPA scale) across different environmental contexts. Lines represent group‐specific regression slopes for each primary school, middle school, district, or municipality, estimated from multilevel random‐slope models. Color gradients reflect the average academic performance of each context (yellow = higher‐performing; purple = lower‐performing). Steeper or more widely dispersed slopes indicate stronger environmental moderation of trait effects, whereas tightly clustered slopes indicate more uniform effects across environments.

Results indicate that a total of 10 out of 16 traits showed significant interactions with at least one environmental level (Table 4; Figure 1). Depression is the only trait among the 16 observed to interact at both district and middle school levels. Five traits (Anxiety, Conduct Disorder, Eating Disorder, Openness, and Autism) showed no significant interaction effects, meaning their impact on GPA was consistent across schools or residential areas.

**TABLE 4:** Best fitted model for each trait.

Category	Trait	Random slope specified level	AIC
Neurodevelopmental Traits	**1. Communication skill problems**	Primary school	64272.95
	**2. Language difficulties**	Middle school	64601.09
	3. Autism	None	65437.82
	**4. ADHD**	Primary school	63163.43
	**5. Attention deficit**	Municipality	62588.34
	**6. Hyperactivity disorder**	Middle school	64614.66
Mental and behavioral traits	**7. Depression**	District, middle school	65111.34
	8. Anxiety	None	65745.52
	**9. Oppositional defiant disorder**	Middle school	65275.83
	10. Conduct disorder	None	64720.91
	11. Eating disorder	None	65703.99
Big five personality traits	12. Openness	None	64502.61
	**13. Conscientiousness**	District	63803.48
	**14. Agreeableness**	District	65433.47
	**15. Extraversion**	Middle school	65730.62
	**16. Neuroticism**	Middle school	65432.04

*Note*: We categorized the traits into three main categories: mental health traits, personality traits, and neurodevelopment traits (John et al., 1991; Morris‐Rosendahl & Crocq, 2020; American Psychiatric Association, 2013) for summary and comparison of the results. Bolded Traits exhibit interactions with environmental contexts. Full results for model comparisons can be found in Supporting Information S1: Table S2.

Overall, more trait‐environment interactions occurred at the school level, particularly in middle schools, than in residential areas. Six out of 16 traits were found to interact at the middle school level, three were found to interact at the district level, two interact at the primary school level and one at the municipality level. Primary schools mainly moderate neurodevelopmental traits, while middle schools moderate a broader range of traits spanning neurodevelopmental traits, mental and behavioral traits, and big five personality traits. Residential contexts, especially districts, also played a significant role for traits like Depression, Conscientiousness, and Agreeableness.

##### Main finding 2: The sizes of psychological trait effects differ by environment and trait category, with the largest slope variance found for neurodevelopmental traits in primary schools

**FIGURE 2:**
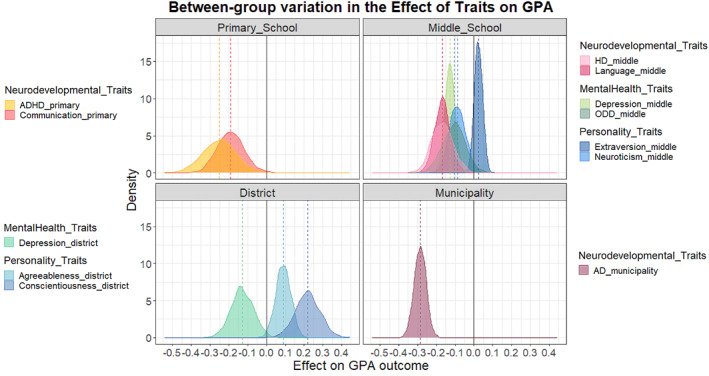
Density Plot of Between‐Group Variation in the Effects of Traits on GPA by Grouping Levels. Density distributions show the estimated random‐slope variation in the association between each psychological trait and students' grade point average (GPA; standardized in the models) across four environmental contexts: primary schools, middle schools, districts, and municipalities. Each colored density represents the distribution of trait effects across units within that level (e.g., across all primary schools for ADHD). Wider densities indicate greater heterogeneity in how strongly a trait predicts GPA across environments, reflecting stronger environmental moderation. Vertical dashed lines mark the mean effect for each trait within the environmental level; the black vertical line denotes zero (no effect). Neurodevelopmental traits show the largest between‐school variation at the primary‐school level, whereas middle schools and districts show smaller but meaningful variation for mental‐health and personality traits.

We quantified the variation of the psychopathology effects on GPA between schools and residential areas. Overall, large variances in the slopes (widths of density in Figure 2) are observed in primary schools, followed by districts, middle schools, and municipalities. Wider variance indicates stronger environmental moderation of trait effects. Narrow variance implies consistent effects regardless of schools/residential areas.

The largest variances of the slopes are found in the neurodevelopmental traits ADHD and Communication Skills at the primary school level with a variance of 0.8% and 0.5% respectively. This indicates that for ADHD, 95% of the primary schools have an effect between −0.077 and −0.428, while the effects are more extreme in the remaining 5%. Similarly for Communication skills, the effects are between −0.055 and −0.331 for 95% of the primary schools. In contrast, Extraversion and Depression in middle schools showed smaller variances (0.05% and 0.07%, respectively). This suggests that these effects on students' GPA are relatively concentrated, varying from −0.019 to 0.068 and −0.076 to −0.18 respectively in 95% of the middle schools. Depression also varied by district (SD = 0.058), suggesting district environments contribute more than twice as much to GPA variation in this trait than middle schools (SD = 0.027).

##### Main finding 3: Psychological trait effects on GPA are weaker in better‐performing schools and residential areas

Across all psychological traits and contexts, stronger average GPA environments were associated with weaker trait effects (Supporting Information S1: Figures S6 and S7). That is, in high‐performing schools or residential areas, students with psychological challenges performed more similarly to peers without such challenges.

As illustrated in Figure 1, this pattern is visually represented by the color‐coded regression lines: yellow lines correspond to high‐performing schools or areas, and purple lines to lower‐performing ones. For example, in the case of neuroticism within middle schools, students with high neuroticism (e.g., 2 standard deviations above the mean) achieve nearly the same GPA as students with low neuroticism (2 SDs below the mean) when they attend higher‐performing (yellow) schools. In contrast, in lower‐performing (purple) schools, students with high neuroticism show markedly lower GPA scores compared to their peers with lower levels of the trait. This pattern suggests high‐performing environments may buffer or compensate for the negative impact of psychological traits on educational performance. In such contexts, individual differences in traits like neuroticism are less predictive of educational performance, implying a protective or equalizing role of high‐performing environments. Conversely, in lower‐performing settings, psychological traits exert a stronger influence on GPA, potentially amplifying existing educational disparities among students.

Notably, for traits such as ADHD, communication skill issues, depression, oppositional defiant disorder (ODD), and conscientiousness, children are with a higher level of psychological challenges (i.e., left side of the crossing point in Figure 1 for Conscientiousness and the opposite for the rest of the traits) perform better if they are from an on average better performing school/residential areas. Whereas for children with a lower level of challenges, children in an academically challenged school/residential area perform better than those who are in an advantaged school/residential area.

##### Main finding 4: School and residential environments differentiate children's performance more when children have a higher level of psychological challenges than the average

**FIGURE 3:**
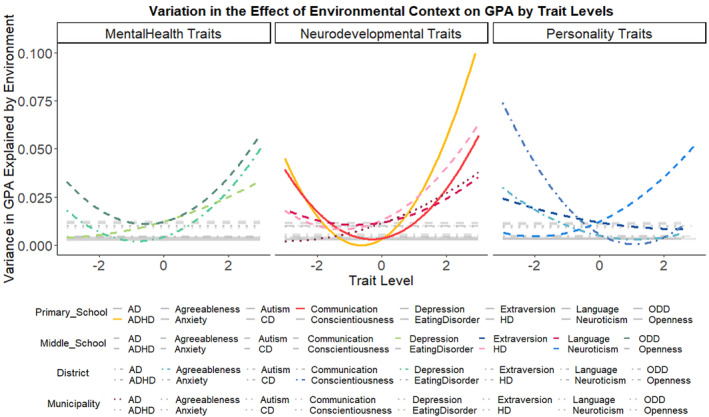
Curves for the variation in the effect of schools and Residential areas by trait measures. AD, Communication, Language, ODD, ADHD, CD, and HD stands for Attention Deficit, Communication skills, Language Difficulties, Oppositional Defiant Disorder, Attention‐Deficit/Hyperactivity Disorder, Conduct Disorder, and Hyperactivity Disorder respectively.

The extent to which school and residential environments influence students' GPA depends on the severity of their psychological traits. Figure 3 illustrates the environmental importance at different levels of the traits, with the *y* axis showing the variance of the average GPA per environmental context, and the *x* axis mapping children's trait level. The larger the variance of the average GPA per environmental context at a given level of trait, the larger impact the environment has on children accordingly.

As shown in Figure 3, most environmental effects follow a U‐shaped pattern, where the influence of the environment is weakest around average trait levels and increases as trait levels become more extreme—particularly on the higher (more severe) end. For example, in the case of ODD, middle schools explain only a small portion (*y* = 1.10%) of GPA variance when students exhibit slightly below‐average levels (*x* = −0.567) of the trait. The environmental effects increase rapidly as the trait levels depart from the bottom on both sides of the scale, however, the right end is stronger than the left end. For instance, middle schools account for 1.86% of GPA variance at −2 SD below average ODD levels, and 3.54% at +2 SD above average, indicating a stronger environmental impact for chilren with more pronounced behavioral challenges. Similar patterns can be found in psychological and neurodevelopmental distress.

Other environmental effects show a monotonical trend with a stronger effect toward the end of a more severe level of trait. For example, Neuroticism and AD (Attention Deficit) demonstrate a steady increase in environmental effect as trait severity grows. Conversely, traits like Extraversion and Conscientiousness exhibit decreasing trends, where the environment matters more for students with lower levels of the trait.

Finally, the gray lines in Figure 3 present the traits that have no interaction with the environments. The shape of the horizontal line throughout the *x*‐axis indicates that the specific environment has the same effect on students' GPA, regardless of the severity of the trait.

## DISCUSSION

By linking Norwegian population‐wide school and geographical data with multidimensional psychological traits, this study provides a comprehensive analysis of how children's educational performance is shaped by the interplay between psychological traits and environmental contexts. Consistent with Bronfenbrenner's ecological model (Bronfenbrenner & Morris, 2007), cross‐layered influences contribute synchronously to school performance, demonstrating that children's development is embedded within interconnected environmental systems. Primary schools, middle schools, districts, and municipalities played crucial roles in moderating the effects of neurodevelopmental, mental health, and personality traits.

Our findings highlight that schools and residential areas function as both protective and risk factors in shaping educational performance. In higher‐performing schools and advantaged districts, children with psychological challenges performed comparably to their peers with fewer difficulties, suggesting patterns consistent with compensatory mechanisms, though we emphasize that these patterns reflect associations rather than causal effects. Conversely, in lower‐performing schools and disadvantaged districts, children with greater psychological challenges faced exacerbated academic struggles. This matches the previous theoretical framework (Reiss et al., 2013; Shanahan & Hofer, 2005) and suggests a focus on the quality of environments and context‐sensitive interventions. Interestingly, conscientiousness emerged as an exception to the general compensatory effect. Children with high conscientiousness excelled even in disadvantaged districts, outperforming peers with similar conscientiousness in more advantaged districts. This suggests that certain personality traits may serve as internal compensatory factors in less supportive environments, warranting further exploration into the resilience mechanisms. However, given the observational design, we interpret these patterns cautiously.

Notably, environmental factors are particularly more important for some children than others. We observed that environmental effects were more pronounced for children at the extremes of psychological risk (i.e., those with severe neurodevelopmental challenges or remarkably low difficulties) compared to children with moderate challenges. For example, while district environments accounted for only 0.22% in GPA for children with an average degree of depression symptoms, its effect was substantially greater, with a variance of 5% in GPA for children with more severe symptoms in depression. These findings suggest children's different susceptability to the environment (Ellis & Boyce, 2011). Tailored educational resources that accommodate children's psychological differences rather than relying on uniform developments show their importance (Tomlinson, 2001).

Among all traits examined, neurodevelopmental traits, particularly ADHD and communication difficulties, showed the strongest interactions with the primary school environments, shaping children's later middle school performance. As these traits are highly visible and often impede learning, they are more easily recognized by teachers, increasing the likelihood of targeted support and engagement with school resources (Daley & Birchwood, 2010; Loe & Feldman, 2007). Differences in the availability of special needs support at school or teacher training in recognizing and managing these challenges may contribute to varying outcomes across schools. These findings align with prior research indicating that well‐resourced schools mitigate the academic risks associated with ADHD (Cheesman, Borgen, et al., 2022; Cheesman, Eilertsen, et al., 2022), highlighting the importance of structured educational support in reducing disparities.

While both schools and residential areas moderated psychological effects, school‐level interactions are stronger, particularly in middle school. This is consistent with our understanding that children acquire knowledge and develop behaviors through interactions with school resources and teachers, directly influencing the GPA received in middle school (Norwegian Agency for Quality Assurance in Education, n.d.). However, residential areas significantly influenced traits related to emotional and behavioral regulation, such as depression, agreeableness, and conscientiousness. These traits link to a wide range of externalizing issues like disinhibition and impulsivity, which often co‐occur with ADHD and conduct disorders (Nigg, 2000), were particularly sensitive to residential environments such as socioeconomic status and violence, reinforcing the broader social influences on children's cognitive abilities (Brook et al., 2011; Kroneman et al., 2004; Leventhal & Brooks‐Gunn, 2000). Although school relates directly to educational performance, the environment outside of school plays a role that cannot be neglected in children's development.

Beyond school and neighborhood effects, we found municipal‐level variation in the impact of attention deficits on educational performance, likely reflecting regional differences in ADHD diagnosis and support systems (Flores et al., 2022; Widding‐Havneraas et al., 2023). Besides, Norway's decentralized governance structure may contribute to these differences, as municipalities exert autonomy in educational and healthcare policies (Kvalsund, 2009). These findings suggest that regional policy variations might influence children's academic outcomes, emphasizing the need for equitable access to psychological and educational support across municipalities.

Most traits only interact with one major context instead of interacting with all the established contexts systematically. This finding suggests a dominant environmental context moderates the relationship between psychological traits and children's educational performance. However, we do not assume that other contexts have zero moderating effect on psychological traits, only that the production of educational performance can best be understood at the level where the interplay between person and environment are the largest.

This study has limitations. First, non‐random selection into schools and residential areas may influence our results. Families often sort into neighborhoods and schools based on socioeconomic, cultural, or child‐specific needs, which might create correlations between children's psychological traits and the environments they enter. Although our cross‐classified multilevel models estimate random effects simultaneously across families, schools, and geographical levels to adjust for clustering at other levels, we cannot rule out residential selection processes or unmeasured factors that influence the choice of schools and residential areas. Thus, the compensatory patterns observed in better‐performing schools should be interpreted as associations rather than causal effects. Future work using quasi‐experimental or within‐family designs should be considered to disentangle contextual effects from selection. Second, all psychological traits were assessed using maternal reports at age 8, which may introduce shared method variance or rater‐specific bias. However, psychological traits and educational outcomes come from different sources, where maternal reports versus teacher‐assigned grades, reducing the risk of same‐rater inflation. Mothers are also uniquely positioned to observe children across a wide range of daily contexts, while not directly involved in providing academic evaluations. Nonetheless, triangulation with teacher or clinical assessments would strengthen future research. Third, residential area identifiers change annually due to policy shifts, and while efforts were made to harmonize these identifiers, tracking changes at more granular levels (e.g., neighborhoods) was not possible. Fourth, while the large sample size ensures statistical power, the results may not be generalizable beyond the MoBa cohort, and findings from Norway's relatively equitable social context may not apply to other countries with different social structures. Finally, the study involved estimating a large number of multilevel models to identify the environmental levels most relevant for the moderation. Although we relied on the AIC that penalizes model complexity and reduces overfitting, this approach does not eliminate all concerns related to multiple testing. Thus, while the consistent patterns we observe are likely to be robust, individual trait‐environment interactions should be interpreted cautiously.

Because the study integrated multiple psychological traits and several nested environmental contexts, the analyses were designed as an exploratory, data‐driven investigation rather than a preregistered hypothesis test. Our goal was to map patterns of environmental moderation across traits and contexts, rather than to evaluate a single predefined hypothesis. Although the study was not preregistered, we implemented a strict model‐fitting strategy based on information‐theoretic criteria (AIC), which constrains researchers' degrees of freedom by prioritizing parsimony and penalizing overfitting. We fully recognize the value of preregistration for hypothesis‐driven studies, particularly to minimize risks of selective reporting or model overfitting. Accordingly, the present findings should be interpreted as hypothesis‐generating, and future work could preregister confirmatory analyses that build upon the exploratory patterns identified here.

## CONCLUSION

This study reveals the role of environments in shaping the educational performance of children with diverse psychological traits. Schools and residential areas do not influence all children equally. Instead, they amplify or mitigate educational performance based on individual psychological profiles. These findings highlight the need for context‐specific educational policies and interventions based on the psychological needs of individuals.

## AUTHOR CONTRIBUTIONS

**Qi Qin:** Conceptualization; methodology; formal analysis; visualization; writing—original draft preparation. **Rosa Cheesman:** Conceptualization; methodology; supervision; writing—review and editing. **Espen Moen Eilertsen:** Conceptualization; methodology; supervision; writing—review and editing. **Eivind Ystrom:** Conceptualization; data curation; funding acquisition; resources; supervision; writing—review and editing.

## CONFLICT OF INTEREST STATEMENT

The authors declare no conflicts of interest.

## ETHICAL CONSIDERATIONS

The Norwegian Mother, Father and Child Cohort Study (MoBa) is supported by the Norwegian Ministry of Health and Care Services and the Ministry of Education and Research. Written informed parental consent was obtained for all participating children. The current study used registry and MoBa data from the project SUBPU. The Department of Psychology, University of Oslo, is responsible for the data handling of SUBPU, for which a Data Protection Impact Assessment (DPIA) has been signed by the Head of Department. The project manager is Eivind Ystrom. The study was approved by the Regional Committees for Medical and Health Research Ethics, Norway on 14 November 2017 (reference number: 2017/2205). SUBPU has formal agreements with MoBa and Statistics Norway for data linkage and usage.

## Supporting information

Supporting Information S1

## Data Availability

Data from the Norwegian Mother, Father and Child Cohort Study and the Medical Birth Registry of Norway used in this study are managed by the national health register holders in Norway (Norwegian Institute of public health) and can be made available to researchers, provided approval from the Regional Committees for Medical and Health Research Ethics (REC), compliance with the EU General Data Protection Regulation (GDPR) and approval from the data owners. The consent given by the participants does not open for storage of data on an individual level in repositories or journals. Researchers who want access to data sets for replication should apply through helsedata.no. Access to data sets requires approval from The Regional Committee for Medical and Health Research Ethics in Norway and an agreement with MoBa. The script for data management and analyses can be found at github repository: https://github.com/qiqin4/EA_P_Environments.
