# Supplementary material for: Children's psychological traits and educational performance: How schools and residential areas moderate how individual traits translate into academic outcomes
Source: JCPP Adv. 2026 Feb 4;6(3):e70100. doi: 10.1002/jcv2.70100 (PMC13338995; doi:10.1002/jcv2.70100)
Supplement: Supplementary file 1 [file JCV2-6-e70100-s001.docx]

**Children’s Psychological Traits and Educational Performance: How Schools and Residential Areas Moderate How Individual Traits Translate into Academic Outcomes**

**Supporting Information**

**Figure S1.** Sample Composition

Sample after linkage (n=26,875)

MoBa Database

Norwegian National Educational Database

Register data from Statistics Norway (SSB)

Primary school ID (n=3,035,087)

Middle school ID (n=1,740,023)

GPA (n=1,381,240)

Family ID (n=446,303)

Geographical ID

(n=3,986,414)

MoBa age 8 (n=43,616)

Linked through the Norwegian ID System

**Figure S2.** Distributions of Original Questionnaire Responses for Each Psychological Trait

*Note.* A_grm_fs, AD, ADHD, C_grm_fs, CCC2, CD, E_grm_fs,_HD, N_grm_fs, O_grm_fs, OD, Språk20 stand for Agreeableness, Attention Deficit, Attention-Deficit / Hyperactivity Disorder, Conscientiousness, Communication skills, Conduct Disorder, Extraversion, Hyperactivity Disorder, Neuroticism, Openness, Oppositional Defiant Disorder, and Language Difficulties respectively.

**Figure S3.** Distributions of Scaled Questionnaire Responses for Each Psychological Trait

*Note.* AD, ADHD, CD, Communication, Communication_nor, HD, OD stand for Attention Deficit, Attention-Deficit / Hyperactivity Disorder, Conduct Disorder, Communication skills, Language Difficulties Extraversion, Hyperactivity Disorder, and Oppositional Defiant Disorder respectively.

**Figure S4.** Distribution of GPA Scores

**Figure S5.** Boxplot for the Number of Children per School and Residential Area

**Figure S6.** Relationship between Average Educational Performance and Strength of the Trait Effect

**Figure S7.** Intercept-Slope Correlations contingent on Psychological Trait

**Table S1.** Extended Model Comparisons for Identifying the Environmental Levels Contributing to GPA Variation

| Model | AIC | BIC | logLik | df |
| --- | --- | --- | --- | --- |
| **Baseline Model** | **65750.70** | **65816.30** | **-32867.35** | **8** |
|  |  |  |  |  |
| Model 1 | 65791.82 | 65849.21 | -32888.91 | 7 |
| Model 2 | 65751.33 | 65808.72 | -32868.66 | 7 |
| Model 3 | 65867.42 | 65924.81 | -32926.71 | 7 |
| Model 4 | 65752.96 | 65810.36 | -32869.48 | 7 |
| **Model 5** | **65750.08** | **65807.47** | **-32868.04** | **7** |
| Model 6 | 66029.84 | 66087.23 | -33007.92 | 7 |
|  |  |  |  |  |
| Model 7 | 66039.21 | 66088.41 | -33013.61 | 6 |
| Model 8 | 66031.29 | 66080.49 | -33009.65 | 6 |
| Model 9 | 65753.49 | 65802.69 | -32870.75 | 6 |
| Model 10 | 66140.01 | 66189.20 | -33064.00 | 6 |
| Model 11 | 65866.67 | 65915.87 | -32927.34 | 6 |
| Model 12 | 65882.56 | 65931.76 | 32935.28 | 6 |
| Model 13 | 66031.11 | 66080.30 | -33009.55 | 6 |
| Model 14 | 65751.93 | 65801.13 | -32869.97 | 6 |
| Model 15 | 65757.70 | 65806.89 | -32872.85 | 6 |
| Model 16 | 65868.12 | 65917.31 | -32928.06 | 6 |
| Model 17 | 66073.16 | 66122.35 | -33030.58 | 6 |
| Model 18 | 65791.49 | 65840.69 | -32889.75 | 6 |
| Model 19 | 65805.63 | 65854.82 | -32896.81 | 6 |
| Model 20 | 65954.11 | 66003.30 | -32971.06 | 6 |
| Model 21 | 65801.44 | 65850.63 | -32894.72 | 6 |
|  |  |  |  |  |
| Model 22 | 66043.69 | 66084.69 | -33016.85 | 5 |
| Model 23 | 66148.75 | 66189.75 | -33069.38 | 5 |
| Model 24 | 66153.14 | 66194.13 | -33071.57 | 5 |
| Model 25 | 65884.21 | 65925.20 | -32937.10 | 5 |
| Model 26 | 66044.46 | 66085.45 | -33017.23 | 5 |
| Model 27 | 66036.72 | 66077.72 | -33013.36 | 5 |
| Model 28 | 65762.95 | 65803.95 | -32876.48 | 5 |
| Model 29 | 66141.36 | 66182.36 | -33065.68 | 5 |
| Model 30 | 65868.52 | 65909.51 | -32929.26 | 5 |
| Model 31 | 65892.04 | 65933.04 | -32941.02 | 5 |
| Model 32 | 66083.20 | 66124.19 | -33036.60 | 5 |
| Model 33 | 66085.37 | 66126.36 | -33037.68 | 5 |
| Model 34 | 65807.51 | 65848.50 | -32898.75 | 5 |
| Model 35 | 66227.80 | 66268.79 | -33108.90 | 5 |
| Model 36 | 65953.69 | 65994.68 | -32971.84 | 5 |
| Model 37 | 66034.67 | 66075.66 | -33012.33 | 5 |
| Model 38 | 66084.57 | 66125.56 | -33037.28 | 5 |
| Model 39 | 65805.31 | 65846.30 | -32897.65 | 5 |
| Model 40 | 65846.71 | 65887.70 | -32918.35 | 5 |
| Model 41 | 65969.17 | 66010.16 | -32979.58 | 5 |
|  |  |  |  |  |
| Model 42 | 66167.69 | 66200.49 | -33079.85 | 4 |
| Model 43 | 66060.69 | 66093.48 | -33026.34 | 4 |
| Model 44 | 66153.84 | 66186.64 | -33072.92 | 4 |
| Model 45 | 66163.49 | 66196.28 | -33077.74 | 4 |
| Model 46 | 65900.92 | 65933.72 | -32946.46 | 4 |
| Model 47 | 66100.63 | 66133.43 | -33046.32 | 4 |
| Model 48 | 66237.15 | 66269.94 | -33114.57 | 4 |
| Model 49 | 66034.67 | 66075.66 | -33012.33 | 4 |
| Model 50 | 66040.08 | 66072.88 | -33016.04 | 4 |
| Model 51 | 66105.07 | 66137.87 | -33048.54 | 4 |
| Model 52 | 66129.01 | 66161.80 | -33060.50 | 4 |
| Model 53 | 65875.71 | 65908.51 | -32933.85 | 4 |
| Model 54 | 66245.23 | 66278.03 | -33118.62 | 4 |
| Model 55 | 65973.88 | 66006.68 | -32982.94 | 4 |
| Model 56 | 66223.44 | 66256.24 | -33107.72 | 4 |
|  |  |  |  |  |
| Model 57 | 66193.34 | 66217.94 | -33093.67 | 3 |
| Model 58 | 66322.18 | 66346.77 | -33158.09 | 3 |
| Model 59 | 66192.48 | 66217.07 | -33093.24 | 3 |
| Model 60 | 66266.72 | 66291.32 | -33130.36 | 3 |
| Model 61 | 66488.69 | 66513.29 | -33241.34 | 3 |
| Model 62 | 66378.94 | 66403.53 | -33186.47 | 3 |

*Note.* This table reports the Akaike Information Criterion (AIC), Bayesian Information Criterion (BIC), log-likelihood (logLik), and degrees of freedom (df) for the baseline model and 62 alternative multilevel models differing in their random-effects structure. Lower AICvalues indicate better relative model fit. **Bolded rows indicate the best-fitting specification (lowest AIC) among all models compared.**

**Table S2.** Model comparison of random‐slope specifications for child psychological traits across contextual levels.

| **Trait** | **Level** | **AIC** | **BIC** | **logLik** | **df** | **Message** |
| --- | --- | --- | --- | --- | --- | --- |
| Communication | municipality, district, primary, middle | 64281.75 | 64412.94 | -32124.88 | 16 | boundary (singular) fit: see help("isSingular") |
| Communication | district, primary, middle | 64284.19 | 64398.97 | -32128.09 | 14 | boundary (singular) fit: see help("isSingular") |
| Communication | municipality, primary, middle | 64282.24 | 64397.03 | -32127.12 | 14 | boundary (singular) fit: see help("isSingular") |
| Communication | municipality, district, middle | 64284.32 | 64399.11 | -32128.16 | 14 | boundary (singular) fit: see help("isSingular") |
| Communication | municipality, district, primary | 64277.82 | 64392.6 | -32124.91 | 14 | boundary (singular) fit: see help("isSingular") |
| Communication | municipality, district | 64280.5 | 64378.89 | -32128.25 | 12 | boundary (singular) fit: see help("isSingular") |
| Communication | municipality, primary | 64274.51 | 64372.9 | -32125.25 | 12 | boundary (singular) fit: see help("isSingular") |
| Communication | municipality, middle | 64288.01 | 64386.4 | -32132.01 | 12 | boundary (singular) fit: see help("isSingular") |
| Communication | district, primary | 64276.07 | 64374.45 | -32126.03 | 12 |  |
| Communication | district, middle | 64282.31 | 64380.7 | -32129.15 | 12 | boundary (singular) fit: see help("isSingular") |
| Communication | primary, middle | 64280.89 | 64379.28 | -32128.44 | 12 | boundary (singular) fit: see help("isSingular") |
| Communication | middle | 64286.27 | 64368.26 | -32133.14 | 10 |  |
| **Communication** | **primary** | **64272.95** | **64354.94** | **-32126.48** | **10** |  |
| Communication | district | 64284.92 | 64366.91 | -32132.46 | 10 | boundary (singular) fit: see help("isSingular") |
| Communication | municipality | 64287.05 | 64369.04 | -32133.53 | 10 | boundary (singular) fit: see help("isSingular") |
| Communication | none | 64286.25 | 64351.84 | -32135.12 | 8 |  |
|  |  |  |  |  |  |  |
| Language Difficulties | municipality, district, primary, middle | 64610.58 | 64741.76 | -32289.29 | 16 | Model failed to converge with max\|grad] = 0.00637082 (tol ... |
| Language Difficulties | district, primary, middle | 64607.981 | 64722.76 | -32289.99 | 14 | Model failed to converge with max\|grad] = 0.00307858 (tol ... |
| Language Difficulties | municipality, primary, middle | 64606.61 | 64721.4 | -32289.31 | 14 | Model failed to converge with max\|grad] = 0.00445699 (tol ... |
| Language Difficulties | municipality, district, middle | 64607.03 | 64721.81 | -32289.51 | 14 | Model failed to converge with max\|grad] = 0.00255694 (tol ... |
| Language Difficulties | municipality, district, primary | 64607.57 | 64722.36 | -32289.79 | 14 | boundary (singular) fit: see help("isSingular") |
| Language Difficulties | municipality, district | 64604.56 | 64702.95 | -32290.28 | 12 | Model failed to converge with max\|grad] = 0.00371079 (tol ... |
| Language Difficulties | municipality, primary | 64603.61 | 64701.99 | -32289.8 | 12 | Model failed to converge with max\|grad] = 0.00483604 (tol ... |
| Language Difficulties | municipality, middle | 64603.35 | 64701.74 | -32289.67 | 12 | Model failed to converge with max\|grad] = 0.00571433 (tol ... |
| Language Difficulties | district, primary | 64610.23 | 64708.62 | -32293.11 | 12 | boundary (singular) fit: see help("isSingular") |
| Language Difficulties | district, middle | 64604.44 | 64702.83 | -32290.22 | 12 | Model failed to converge with max\|grad] = 0.00289466 (tol ... |
| Language Difficulties | primary, middle | 64608.49 | 64706.87 | -32292.24 | 12 | boundary (singular) fit: see help("isSingular") |
| **Language Difficulties** | **middle** | **64601.09** | **64683.08** | **-32290.55** | **10** |  |
| Language Difficulties | primary | 64602.11 | 64684.1 | -32291.06 | 10 |  |
| Language Difficulties | district | 64602.89 | 64684.88 | -32291.45 | 10 |  |
| Language Difficulties | municipality | 64602.25 | 64684.24 | -32291.13 | 10 | Model failed to converge with max\|grad] = 0.00374476 (tol ... |
| Language Difficulties | none | 64602.85 | 64668.44 | -32293.42 | 8 |  |
|  |  |  |  |  |  |  |
| Autism | municipality, district, primary, middle | 65416.55 | 65547.74 | -32692.28 | 16 | boundary (singular) fit: see help("isSingular") |
| Autism | district, primary, middle | 65403.25 | 65518.04 | -32687.63 | 14 | boundary (singular) fit: see help("isSingular") |
| Autism | municipality, primary, middle | 65402.24 | 65517.02 | -32687.12 | 14 | boundary (singular) fit: see help("isSingular") |
| Autism | municipality, district, middle | 65418.61 | 65533.39 | -32695.3 | 14 | boundary (singular) fit: see help("isSingular") |
| Autism | municipality, district, primary | 65406.59 | 65521.38 | -32689.3 | 14 | boundary (singular) fit: see help("isSingular") |
| Autism | municipality, district | 65414.64 | 65513.03 | -32695.32 | 12 | boundary (singular) fit: see help("isSingular") |
| Autism | municipality, primary | 65398.31 | 65496.7 | -32687.16 | 12 | boundary (singular) fit: see help("isSingular") |
| Autism | municipality, middle | 65435.89 | 65534.28 | -32705.95 | 12 | boundary (singular) fit: see help("isSingular") |
| Autism | district, primary | 65399.27 | 65497.65 | -32687.63 | 12 | boundary (singular) fit: see help("isSingular") |
| Autism | district, middle | 65417.34 | 65515.73 | -32696.67 | 12 | Model failed to converge with maxigral = 0.00822988 (tol... |
| Autism | primary, middle | 65416.94 | 65515.33 | -32696.47 | 12 | boundary (singular) fit: see help("isSingular") |
| Autism | middle | 65434.42 | 65516.41 | -32707.21\| | 10 | Model failed to converge with max\|grad] = 0.00346771 (tol ... |
| Autism | primary | 65396.66 | 65478.65 | -32688.33 | 10 | boundary (singular) fit: see help("isSingular") |
| Autism | district | 65423.39 | 65505.38 | -32701.7 | 10 | boundary (singular) fit: see help("isSingular") |
| Autism | municipality | 65438.62 | 65520.61 | -32709.31 | 10 | Model failed to converge with max/grad] = 0.00903573 (tol ... |
| **Autism** | **none** | **65437.82** | **65503.41** | **-32710.91** | **8** |  |
|  |  |  |  |  |  |  |
| Depression | municipality, district, primary, middle | 65108.49 | 65239.68 | -32538.25 | 16 | boundary (singular) fit: see help("isSingular") |
| Depression | district, primary, middle | 65108.87 | 65223.66 | -32540.44 | 14 | boundary (singular) fit: see help("isSingular") |
| Depression | municipality, primary, middle | 65112.62 | 65227.41 | -32542.31 | 14 | boundary (singular) fit: see help("isSingular") |
| Depression | municipality, district, middle | 65121.11 | 65235.9 | -32546.56 | 14 | boundary (singular) fit: see help("isSingular") |
| Depression | municipality, district, primary | 65106.33 | 65221.11 | -32539.16 | 14 | boundary (singular) fit: see help("isSingular") |
| Depression | municipality, district | 65110.08 | 65208.46 | -32543.04 | 12 | boundary (singular) fit: see help("isSingular") |
| Depression | municipality, primary | 65105.64 | 65204.03 | -32540.82 | 12 | boundary (singular) fit: see help("isSingular") |
| Depression | municipality, middle | 65116.6 | 65214.99 | -32546.3 | 12 | boundary (singular) fit: see help("isSingular") |
| Depression | district, primary | 65107.88 | 65206.27 | -32541.94 | 12 | boundary (singular) fit: see help("isSingular") |
| **Depression** | **district, middle** | **65111.34** | **65209.72** | **-32543.67** | **12** |  |
| Depression | primary, middle | 65113.85 | 65212.24 | -32544.93 | 12 | boundary (singular) fit: see help("isSingular") |
| Depression | middle | 65117.52 | 65199.51 | -32548.76 | 10 | Model failed to converge with max\|grad] = 0.00317146 (tol ... |
| Depression | primary | 65107.93 | 65189.92 | -32543.97 | 10 | Model failed to converge with max\|grad] = 0.00655052 (tol ... |
| Depression | district | 65111.61 | 65193.6 | -32545.81 | 10 | unable to evaluate scaled gradient; Model failed to converg... |
| Depression | municipality | 65125.93 | 65207.91 | -32552.96 | 10 | Model failed to converge with max\|grad] = 0.00255435 (tol ... |
| Depression | none | 65133.11 | 65198.7 | -32558.55 | 8 |  |
|  |  |  |  |  |  |  |
| Anxiety | municipality, district, primary, middle | 65747.71 | 65878.9 | -32857.86 | 16 | boundary (singular) fit: see help("isSingular") |
| Anxiety | district, primary, middle | 65749.62 | 65864.4 | -32860.81 | 14 | boundary (singular) fit: see help("isSingular") |
| Anxiety | municipality, primary, middle | 65743.8 | 65858.58 | -32857.9 | 14 | boundary (singular) fit: see help("isSingular") |
| Anxiety | municipality, district, middle | 65746.24 | 65861.02 | -32859.12 | 14 | boundary (singular) fit: see help("isSingular") |
| Anxiety | municipality, district, primary | 65758.69 | 65873.47 | -32865.34 | 14 | boundary (singular) fit: see help("isSingular") |
| Anxiety | municipality, district | 65751.86 | 65850.24 | -32863.93 | 12 | boundary (singular) fit: see help("isSingular") |
| Anxiety | municipality, primary | 65745.72 | 65844.1 | -32860.86 | 12 | boundary (singular) fit: see help("isSingular") |
| Anxiety | municipality, middle | 65742.24 | 65840.631 | -32859.12 | 12 | boundary (singular) fit: see help("isSingular") |
| Anxiety | district, primary | 65745.78 | 65844.16 | -32860.89 | 12 | boundary (singular) fit: see help("isSingular") |
| Anxiety | district, middle | 65742.26 | 65840.64 | -32859.13 | 12 | boundary (singular) fit: see help("isSingular") |
| Anxiety | primary, middle | 65739.8 | 65838.18 | -32857.9 | 12 | boundary (singular) fit: see help("isSingular") |
| Anxiety | middle | 65738.26 | 65820.25 | -32859.13 | 10 | boundary (singular) fit: see help("isSingular") |
| Anxiety | primary | 65752.07 | 65834.06 | -32866.03 | 10 | boundary (singular) fit: see help("isSingular") |
| Anxiety | district | 65748.19 | 65830.18 | -32864.1 | 10 | boundary (singular) fit: see help("isSingular") |
| Anxiety | municipality | 65749.01 | 65831 | -32864.5 | 10 | boundary (singular) fit: see help("isSingular") |
| **Anxiety** | **none** | **65745.52** | **65811.11** | **-32864.76** | **8** |  |
|  |  |  |  |  |  |  |
| Eating Disorder | municipality, district, primary, middle | 65712.64 | 65843.83 | -32840.32 | 16 | boundary (singular) fit: see help("isSingular") |
| Eating Disorder | district, primary, middle | 65711.97 | 65826.76 | -32841.99 | 14 | boundary (singular) fit: see help("isSingular") |
| Eating Disorder | municipality, primary, middle | 65708.91 | 65823.7 | -32840.46 | 14 | boundary (singular) fit: see help("isSingular") |
| Eating Disorder | municipality, district, middle | 65710.87 | 65825.65 | -32841.43 | 14 | boundary (singular) fit: see help("isSingular") |
| Eating Disorder | municipality, district, primary | 65709.48 | 65824.26 | -32840.74 | 14 | boundary (singular) fit: see help("isSingular") |
| Eating Disorder | municipality, district | 65707.3 | 65805.69 | -32841.65 | 12 | boundary (singular) fit: see help("isSingular") |
| Eating Disorder | municipality, primary | 65705.59 | 65803.97 | -32840.79 | 12 | boundary (singular) fit: see help("isSingular") |
| Eating Disorder | municipality, middle | 65707.97 | 65806.36 | -32841.99 | 12 | boundary (singular) fit: see help("isSingular") |
| Eating Disorder | district, primary | 65708.39 | 65806.77 | -32842.19 | 12 | boundary (singular) fit: see help("isSingular") |
| Eating Disorder | district, middle | 65710.43 | 65808.81 | -32843.211 | 12 | boundary (singular) fit: see help("isSingular") |
| Eating Disorder | primary, middle | 65708.41 | 65806.79 | -32842.2 | 12 | boundary (singular) fit: see help("isSingular") |
| Eating Disorder | middle | 65707.99 | 65789.98 | -32844 | 10 | boundary (singular) fit: see help("isSingular") |
| Eating Disorder | primary | 65704.66 | 65786.65 | -32842.33 | 10 | boundary (singular) fit: see help("isSingular") |
| Eating Disorder | district | 65706.55 | 65788.54 | -32843.28 | 10 | boundary (singular) fit: see help("isSingular") |
| Eating Disorder | municipality | 65704.12 | 65786.11 | -32842.06 | 10 | boundary (singular) fit: see help("isSingular") |
| **Eating Disorder** | **none** | **65703.99** | **65769.59** | **-32844** | **8** |  |
|  |  |  |  |  |  |  |
| Conduct Disorder | municipality, district, primary, middle | 64692.2 | 64823.39 | -32330.1 | 16 | boundary (singular) fit: see help("isSingular") |
| Conduct Disorder | district, primary, middle | 64693.67 | 64808.45 | -32332.83 | 14 | boundary (singular) fit: see help("isSingular") |
| Conduct Disorder | municipality, primary, middle | 64688.21 | 64803 | -32330.11 | 14 | Model failed to converge with max\|grad] = 0.00417459 (tol ... |
| Conduct Disorder | municipality, district, middle | 64698.47 | 64813.25 | -32335.23 | 14 | boundary (singular) fit: see help("isSingular") |
| Conduct Disorder | municipality, district, primary | 64691.07 | 64805.86 | -32331.54 | 14 | Model failed to converge with max\|grad] = 0.00460479 (tol ... |
| Conduct Disorder | municipality, district | 64698.63 | 64797.02 | -32337.31 | 12 | boundary (singular) fit: see help("isSingular") |
| Conduct Disorder | municipality, primary | 64687.2 | 64785.59 | -32331.6 | 12 | boundary (singular) fit: see help("isSingular") |
| Conduct Disorder | municipality, middle | 64701.18 | 64799.57 | -32338.59 | 12 | Model failed to converge with max\|grad] = 0.00534566 (tol ... |
| Conduct Disorder | district, primary | 64699.61 | 64797.99 | -32337.8 | 12 | boundary (singular) fit: see help("isSingular") |
| Conduct Disorder | district, middle | 64699.47 | 64797.85 | -32337.73 | 12 | boundary (singular) fit: see help("isSingular") |
| Conduct Disorder | primary, middle | 64690.09 | 64788.48 | -32333.04 | 12 | Model failed to converge with max\|grad] = 0.00978181 (tol ... |
| Conduct Disorder | middle | 64705.21 | 64787.2 | -32342.6 | 10 | Model failed to converge with max\|grad] = 0.00285491 (tol ... |
| Conduct Disorder | primary | 64691.31 | 64773.3 | -32335.66 | 10 | Model failed to converge with max\|grad] = 0.00459521 (tol ... |
| Conduct Disorder | district | 64712.61 | 64794.6 | -32346.3 | 10 | boundary (singular) fit: see help("isSingular") |
| Conduct Disorder | municipality | 64706.35 | 64788.34 | -32343.17 | 10 | Model failed to converge with max\|grad] = 0.00391215 (tol ... |
| **Conduct Disorder** | **none** | **64720.91** | **64786.51** | **-32352.46** | **8** |  |
|  |  |  |  |  |  |  |
| Oppositional Defiant Disorder | municipality, district, primary, middle | 65246.24 | 65377.42 | -32607.12 | 16 | boundary (singular) fit: see help("isSingular") |
| Oppositional Defiant Disorder | district, primary, middle | 65244.81 | 65359.59 | -32608.4 | 14 | Model failed to converge with maxlgrad\| = 0.00566589 (tol.. |
| Oppositional Defiant Disorder | municipality, primary, middle | 65245.25 | 65360.03 | -32608.62 | 14 | boundary (singular) fit: see help("isSingular") |
| Oppositional Defiant Disorder | municipality, district, middle | 65256.65 | 65371.44 | -32614.33 | 14 | Model failed to converge with maxlgrad\| = 0.00793036 (tol ... |
| Oppositional Defiant Disorder | municipality, district, primary | 65249.26 | 65364.05 | -32610.63 | 14 | boundary (singular) fit: see help("isSingular") |
| Oppositional Defiant Disorder | municipality, district | 65252.9 | 65351.28 | -32614.45 | 12 | Model failed to converge with maxlgrad\| = 0.0117805 (tol=... |
| Oppositional Defiant Disorder | municipality, primary | 65241.25 | 65339.64 | -32608.63 | 12 | boundary (singular) fit: see help("isSingular") |
| Oppositional Defiant Disorder | municipality, middle | 65275.02 | 65373.41 | -32625.51 | 12 | Model failed to converge with maxlgrad\| = 0.00542596 (tol ... |
| Oppositional Defiant Disorder | district, primary | 65248.89 | 65347.28 | -32612.44 | 12 | boundary (singular) fit: see help("isSingular") |
| Oppositional Defiant Disorder | district, middle | 65255.06 | 65353.45 | -32615.53 | 12 | Model failed to converge with maxlgrad\| = 0.00269464 (tol ... |
| Oppositional Defiant Disorder | primary, middle | 65255.92 | 65354.31 | -32615.96 | 12 | boundary (singular) fit: see help("isSingular") |
| **Oppositional Defiant Disorder** | **middle** | **65275.83** | **65357.82** | **-32627.92** | **10** |  |
| Oppositional Defiant Disorder | primary | 65254.41 | 65336.4 | -32617.2 | 10 | boundary (singular) fit: see help("isSingular") |
| Oppositional Defiant Disorder | district | 65264.33 | 65346.32 | -32622.17 | 10 | boundary (singular) fit: see help("isSingular") |
| Oppositional Defiant Disorder | municipality | 65280.92 | 65362.91 | -32630.46 | 10 | Model failed to converge with max/grad] = 0.00373417 (tol ... |
| Oppositional Defiant Disorder | none | 65288.98 | 65354.57 | -32636.49 | 8 |  |
|  |  |  |  |  |  |  |
| ADHD | municipality, district, primary, middle | 63155.3 | 63286.49 | -31561.65 | 16 | boundary (singular) fit: see help("isSingular") |
| ADHD | district, primary, middle | 63164.46 | 63279.25 | -31568.23 | 14 | boundary (singular) fit: see help("isSingular") |
| ADHD | municipality, primary, middle | 63164.08 | 63278.86 | -31568.04 | 14 | boundary (singular) fit: see help("isSingular") |
| ADHD | municipality, district, middle | 63174.28 | 63289.06 | -31573.14 | 14 | boundary (singular) fit: see help("isSingular") |
| ADHD | municipality, district, primary | 63152.48 | 63267.27 | -31562.24 | 14 | boundary (singular) fit: see help("isSingular") |
| ADHD | municipality, district | 63186.18 | 63284.57 | -31581.09 | 12 | boundary (singular) fit: see help("isSingular") |
| ADHD | municipality, primary | 63149.47 | 63247.86 | -31562.74 | 12 | boundary (singular) fit: see help("isSingular") |
| ADHD | municipality, middle | 63184.05 | 63282.44 | -31580.03 | 12 | boundary (singular) fit: see help("isSingular") |
| ADHD | district, primary | 63163.3 | 63261.68 | -31569.65 | 12 | Model failed to converge with max\|grad] = 0.0286838 (tol =... |
| ADHD | district, middle | 63182.58 | 63280.97 | -31579.29 | 12 | Model failed to converge with max\|grad] = 0.00442908 (tol ... |
| ADHD | primary, middle | 63164.53 | 63262.92 | -31570.27 | 12 |  |
| ADHD | middle | 63256.91 | 63338.9 | -31618.45 | 10 | boundary (singular) fit: see help("isSingular") |
| **ADHD** | **primary** | **63163.43** | **63245.41** | **-31571.71** | **10** |  |
| ADHD | district | 63199.37 | 63281.36 | -31589.69 | 10 | boundary (singular) fit: see help("isSingular") |
| ADHD | municipality | 63194.63 | 63276.62 | -31587.31 | 10 |  |
| ADHD | none | 63225.2 | 63290.79 | -31604.6 | 8 |  |
|  |  |  |  |  |  |  |
| Attention Deficit | municipality, district, primary, middle | 62566.34 | 62697.52 | -31267.17 | 16 | boundary (singular) fit: see help("isSingular") |
| Attention Deficit | district, primary, middle | 62571.38 | 62686.16 | -31271.69 | 14 | boundary (singular) fit: see help("isSingular") |
| Attention Deficit | municipality, primary, middle | 62555.51 | 62670.3 | -31263.76 | 14 | boundary (singular) fit: see help("isSingular") |
| Attention Deficit | municipality, district, middle | 62574.05 | 62688.84 | -31273.03 | 14 | boundary (singular) fit: see help("isSingular") |
| Attention Deficit | municipality, district, primary | 62563.79 | 62678.58 | -31267.89 | 14 | boundary (singular) fit: see help("isSingular") |
| Attention Deficit | municipality, district | 62571.9 | 62670.29 | -31273.95 | 12 | boundary (singular) fit: see help("isSingular") |
| Attention Deficit | municipality, primary | 62552.12 | 62650.5 | -31264.06 | 12 | boundary (singular) fit: see help("isSingular") |
| Attention Deficit | municipality, middle | 62581.45 | 62679.84 | -31278.73 | 12 | unable to evaluate scaled gradient; Model failed to converg... |
| Attention Deficit | district, primary | 62569 | 62667.39 | 31272.5 | 12 | Model failed to converge with maxgradl = 0.00495086 (tol ... |
| Attention Deficit | district, middle | 62585.42 | 62683.8 | -31280.71 | 12 | Model failed to converge with max/grad] = 0.00226605 (tol ... |
| Attention Deficit | primary, middle | 62570.77 | 62669.16 | -31273.38 | 12 | boundary (singular) fit: see help("isSingular") |
| Attention Deficit | middle | 62600.32 | 62682.31 | -31290.16 | 10 |  |
| Attention Deficit | primary | 62568.84 | 62650.83 | -31274.42 | 10 | Model failed to converge with max\|grad] = 0.00312145 (tol ... |
| Attention Deficit | district | 62585.07 | 62667.06 | -31282.54 | 10 | Model failed to converge with maxgradl = 0.00389868 (tol ... |
| **Attention Deficit** | **municipality** | **62588.34** | **62670.33** | **-31284.17** | **10** |  |
| Attention Deficit | none | 62621.77 | 62687.36 | -31302.89 | 8 |  |
|  |  |  |  |  |  |  |
| Hyperactivity Disorder | municipality, district, primary, middle | 64584.06 | 64715.24 | -32276.03 | 16 | boundary (singular) fit: see help("isSingular") |
| Hyperactivity Disorder | district, primary, middle | 64605.69 | 64720.48 | -32288.85 | 14 | boundary (singular) fit: see help("isSingular") |
| Hyperactivity Disorder | municipality, primary, middle | 64584.22 | 64699 | -32278.11 | 14 | boundary (singular) fit: see help("isSingular") |
| Hyperactivity Disorder | municipality, district, middle | 64596.37 | 64711.15 | -32284.18 | 14 | boundary (singular) fit: see help("isSingular") |
| Hyperactivity Disorder | municipality, district, primary | 64599.61 | 64714.39 | -32285.8 | 14 | boundary (singular) fit: see help("isSingular") |
| Hyperactivity Disorder | municipality, district | 64597.31 | 64695.7 | -32286.66 | 12 | boundary (singular) fit: see help("isSingular") |
| Hyperactivity Disorder | municipality, primary | 64581.47 | 64679.86 | -32278.74 | 12 | Model failed to converge with max\|grad] = 0.0187425 (tol =... |
| Hyperactivity Disorder | municipality, middle | 64603.9 | 64702.29 | -32289.95 | 12 | Model failed to converge with max\|grad] = 0.00340563 (tol ... |
| Hyperactivity Disorder | district, primary | 64590.06 | 64688.45 | -32283.03 | 12 | boundary (singular) fit: see help("isSingular") |
| Hyperactivity Disorder | district, middle | 64602.19 | 64700.57 | -32289.09 | 12 | boundary (singular) fit: see help("isSingular") |
| Hyperactivity Disorder | primary, middle | 64591.73 | 64690.12 | -32283.87 | 12 | boundary (singular) fit: see help("isSingular") |
| **Hyperactivity Disorder** | **middle** | **64614.66** | **64696.65** | **-32297.33** | **10** |  |
| Hyperactivity Disorder | primary | 64591.2 | 64673.19 | -32285.6 | 10 | boundary (singular) fit: see help("isSingular") |
| Hyperactivity Disorder | district | 64615.44 | 64697.43 | -32297.72 | 10 | boundary (singular) fit: see help("isSingular") |
| Hyperactivity Disorder | municipality | 64609.19 | 64691.18 | -32294.59 | 10 | Model failed to converge with max\|grad] = 0.00510123 (tol ... |
| Hyperactivity Disorder | none | 64631.63 | 64697.22 | -32307.81 | 8 |  |
|  |  |  |  |  |  |  |
| Extraversion | municipality, district, primary, middle | 65739.6 | 65870.78 | -32853.8 | 16 | boundary (singular) fit: see help("isSingular") |
| Extraversion | district, primary, middle | 65738.11 | 65852.9 | -32855.06 | 14 | boundary (singular) fit: see help("isSingular") |
| Extraversion | municipality, primary, middle | 65736.1 | 65850.89 | -32854.05 | 14 | boundary (singular) fit: see help("isSingular") |
| Extraversion | municipality, district, middle | 65735.8 | 65850.58 | -32853.9 | 14 | boundary (singular) fit: see help("isSingular") |
| Extraversion | municipality, district, primary | 65736.52 | 65851.31 | -32854.26 | 14 | boundary (singular) fit: see help("isSingular") |
| Extraversion | municipality, district | 65732.99 | 65831.38 | -32854.49 | 12 | boundary (singular) fit: see help("isSingular") |
| Extraversion | municipality, primary | 65732.86 | 65831.24 | -32854.43 | 12 | boundary (singular) fit: see help("isSingular") |
| Extraversion | municipality, middle | 65732.15 | 65830.54 | -32854.08 | 12 | boundary (singular) fit: see help("isSingular") |
| Extraversion | district, primary | 65735.87 | 65834.25 | -32855.93 | 12 | boundary (singular) fit: see help("isSingular") |
| Extraversion | district, middle | 65734.37 | 65832.76 | -32855.19 | 12 | Model failed to converge with max/grad = 0.00440257 (tol ... |
| Extraversion | primary, middle | 65734.52 | 65832.9 | -32855.26 | 12 | boundary (singular) fit: see help("isSingular") |
| **Extraversion** | **middle** | **65730.62** | **65812.61** | **-32855.31** | **10** |  |
| Extraversion | primary | 65732.04 | 65814.03 | -32856.02 | 10 | boundary (singular) fit: see help("isSingular") |
| Extraversion | district | 65732.62 | 65814.61 | -32856.31 | 10 | boundary (singular) fit: see help("isSingular") |
| Extraversion | municipality | 65729.1 | 65811.09 | -32854.55 | 10 | Model failed to converge with max\|grad] = 0.0204637 (tol =... |
| Extraversion | none | 65728.62 | 65794.22 | -32856.31 | 8 | Model failed to converge with max/grad\| = 0.00545547 (tol ... |
|  |  |  |  |  |  |  |
| Agreeableness | municipality, district, primary, middle | 65444.54 | 65575.72 | -32706.27 | 16 | boundary (singular) fit: see help("isSingular") |
| Agreeableness | district, primary, middle | 65433.3 | 65548.08 | -32702.65 | 14 | boundary (singular) fit: see help("isSingular") |
| Agreeableness | municipality, primary, middle | 65433.09 | 65547.87 | -32702.54 | 14 | boundary (singular) fit: see help("isSingular") |
| Agreeableness | municipality, district, middle | 65445.88 | 65560.67 | -32708.94 | 14 | boundary (singular) fit: see help("isSingular") |
| Agreeableness | municipality, district, primary | 65433.88 | 65548.66 | -32702.94 | 14 | boundary (singular) fit: see help("isSingular") |
| Agreeableness | municipality, district | 65437.08 | 65535.46 | -32706.54 | 12 | boundary (singular) fit: see help("isSingular") |
| Agreeableness | municipality, primary | 65440.31 | 65538.7 | -32708.15 | 12 | boundary (singular) fit: see help("isSingular") |
| Agreeableness | municipality, middle | 65435.44 | 65533.83 | -32705.72 | 12 | boundary (singular) fit: see help("isSingular") |
| Agreeableness | district, primary | 65430.23 | 65528.62 | -32703.11 | 12 | boundary (singular) fit: see help("isSingular") |
| Agreeableness | district, middle | 65434.79 | 65533.18 | -32705.4 | 12 |  |
| Agreeableness | primary, middle | 65437.81 | 65536.2 | -32706.9 | 12 | boundary (singular) fit: see help("isSingular") |
| Agreeableness | middle | 65431.74 | 65513.73 | -32705.87 | 10 | Model failed to converge with max\|grad] = 0.00226414 (tol ... |
| Agreeableness | primary | 65426.25 | 65508.24 | -32703.12 | 10 | boundary (singular) fit: see help("isSingular") |
| **Agreeableness** | **district** | **65433.47** | **65515.46** | **-32706.74** | **10** |  |
| Agreeableness | municipality | 65437.16 | 65519.15 | -32708.58 | 10 | Model failed to converge with max\|grad] = 0.0101154 (tol =... |
| Agreeableness | none | 65434.25 | 65499.84 | -32709.13 | 8 |  |
|  |  |  |  |  |  |  |
| Conscientiousness | municipality, district, primary, middle | 63801.07 | 63932.26 | -31884.54 | 16 | boundary (singular) fit: see help("isSingular") |
| Conscientiousness | district, primary, middle | 63806.93 | 63921.71 | -31889.46 | 14 | boundary (singular) fit: see help("isSingular") |
| Conscientiousness | municipality, primary, middle | 63804.72 | 63919.5 | -31888.36 | 14 | boundary (singular) fit: see help("isSingular") |
| Conscientiousness | municipality, district, middle | 63797.38 | 63912.17 | -31884.69 | 14 | boundary (singular) fit: see help("isSingular") |
| Conscientiousness | municipality, district, primary | 63798.82 | 63913.61 | -31885.41\| | 14 | boundary (singular) fit: see help("isSingular") |
| Conscientiousness | municipality, district | 63795.23 | 63893.62 | -31885.62 | 12 | boundary (singular) fit: see help("isSingular") |
| Conscientiousness | municipality, primary | 63804.07 | 63902.46 | -31890.04 | 12 | boundary (singular) fit: see help("isSingular") |
| Conscientiousness | municipality, middle | 63804.28 | 63902.67 | -31890.14 | 12 | Model failed to converge with max/grad\| = 0.00301167 (tol ... |
| Conscientiousness | district, primary | 63806.55 | 63904.94 | -31891.281 | 12 | Model failed to converge with max\|grad\| = 0.00802803 (tol ... |
| Conscientiousness | district, middle | 63823.33 | 63921.71 | -31899.66 | 12 | boundary (singular) fit: see help("isSingular") |
| Conscientiousness | primary, middle | 63813.21 | 63911.6 | -31894.61 | 12 | boundary (singular) fit: see help("isSingular") |
| Conscientiousness | middle | 63814.3 | 63896.29 | -31897.15 | 10 | Model failed to converge with max/grad\| = 0.0230863 (tol =... |
| Conscientiousness | primary | 63818.16 | 63900.15 | -31899.081 | 10 | boundary (singular) fit: see help("isSingular") |
| **Conscientiousness** | **district** | **63803.48** | **63885.47** | **-31891.74** | **10** |  |
| Conscientiousness | municipality | 63807.53 | 63889.52 | -31893.76 | 10 |  |
| Conscientiousness | none | 63831.23 | 63896.82 | -31907.61 | 8 |  |
|  |  |  |  |  |  |  |
| Neuroticism | municipality, district, primary, middle | 65438.09 | 65569.27 | -32703.04 | 16 | boundary (singular) fit: see help("isSingular") |
| Neuroticism | district, primary, middle | 65436.49 | 65551.28 | -32704.25 | 14 | boundary (singular) fit: see help("isSingular") |
| Neuroticism | municipality, primary, middle | 65434.84 | 65549.63 | -32703.42 | 14 | boundary (singular) fit: see help("isSingular") |
| Neuroticism | municipality, district, middle | 65435.54 | 65550.33 | -32703.77 | 14 | boundary (singular) fit: see help("isSingular") |
| Neuroticism | municipality, district, primary | 65439.88 | 65554.66 | -32705.94 | 14 | boundary (singular) fit: see help("isSingular") |
| Neuroticism | municipality, district | 65439.18 | 65537.57 | -32707.59 | 12 | boundary (singular) fit: see help("isSingular") |
| Neuroticism | municipality, primary | 65438.03 | 65536.42 | -32707.02 | 12 | boundary (singular) fit: see help("isSingular") |
| Neuroticism | municipality, middle | 65433.23 | 65531.62 | -32704.62 | 12 | boundary (singular) fit: see help("isSingular") |
| Neuroticism | district, primary | 65440.11 | 65538.49 | -32708.05 | 12 | boundary (singular) fit: see help("isSingular") |
| Neuroticism | district, middle | 65434.1 | 65532.49 | -32705.05 | 12 | boundary (singular) fit: see help("isSingular") |
| Neuroticism | primary, middle | 65433.35 | 65531.74 | -32704.68 | 12 | boundary (singular) fit: see help("isSingular") |
| **Neuroticism** | **middle** | **65432.04** | **65514.03** | **-32706.02** | **10** |  |
| Neuroticism | primary | 65439.03 | 65521.02 | -32709.51 | 10 | boundary (singular) fit: see help("isSingular") |
| Neuroticism | district | 65440.14 | 65522.13 | -32710.07 | 10 | boundary (singular) fit: see help("isSingular") |
| Neuroticism | municipality | 65441.83 | 65523.82 | -32710.92 | 10 | Model failed to converge with max/grad] = 0.00361036 (tol ... |
| Neuroticism | none | 65445.65 | 65511.24 | -32714.82 | 8 |  |
|  |  |  |  |  |  |  |
| Openness | municipality, district, primary, middle | 64506.08 | 64637.26 | -32237.04 | 16 | boundary (singular) fit: see help("isSingular") |
| Openness | district, primary, middle | 64509.13 | 64623.91 | -32240.56 | 14 | boundary (singular) fit: see help("isSingular") |
| Openness | municipality, primary, middle | 64502.53 | 64617.31 | -32237.26 | 14 | boundary (singular) fit: see help("isSingular") |
| Openness | municipality, district, middle | 64502.25 | 64617.03 | -32237.12 | 14 | boundary (singular) fit: see help("isSingular") |
| Openness | municipality, district, primary | 64503.8 | 64618.58 | -32237.9 | 14 | Model failed to converge with max\|grad\| = 0.0194015 (tol =... |
| Openness | municipality, district | 64499.96 | 64598.35 | -32237.98 | 12 | Model failed to converge with max\|grad] = 0.0022202 (tol =... |
| Openness | municipality, primary | 64503.02 | 64601.41 | -32239.51 | 12 | boundary (singular) fit: see help("isSingular") |
| Openness | municipality, middle | 64498.98 | 64597.37 | -32237.49 | 12 | boundary (singular) fit: see help("isSingular") |
| Openness | district, primary | 64508.44 | 64606.83 | -32242.22 | 12 | Model failed to converge with max\|grad] = 0.00595781 (tol ... |
| Openness | district, middle | 64513.42 | 64611.81 | -32244.71 | 12 | boundary (singular) fit: see help("isSingular") |
| Openness | primary, middle | 64505.81 | 64604.2 | -32240.91 | 12 | Model failed to converge with max\|grad] = 0.00885415 (tol ... |
| Openness | middle | 64502.85 | 64584.84 | -32241.43 | 10 |  |
| Openness | primary | 64508.23 | 64590.22 | -32244.11 | 10 | boundary (singular) fit: see help("isSingular") |
| Openness | district | 64504.83 | 64586.82 | -32242.42 | 10 |  |
| Openness | municipality | 64496.19 | 64578.18 | -32238.09 | 10 | Model failed to converge with max\|grad] = 0.00362346 (tol ... |
| **Openness** | **none** | **64502.61** | **64568.2** | **-32243.3** | **8** |  |
|  |  |  |  |  |  |  |

*Note.* For each trait, multilevel linear mixed models predicting standardized grade point average were fitted with random slopes at different combinations of municipality, district, primary school, and lower secondary school levels. The table reports, for every trait–level combination, the Akaike Information Criterion (AIC), Bayesian Information Criterion (BIC), log-likelihood (logLik), and model degrees of freedom (df). The Level column indicates the set of levels at which trait slopes were allowed to vary, with “none” denoting a random-intercept–only model. The Message column reproduces lme4 optimisation warnings (e.g. boundary/singular fits or non-convergence) used to flag unstable models. Bolded rows indicate the best-fitting model for each trait (lowest AIC) among the random-slope specifications considered.
